# *Strongyloides* infection manifested during immunosuppressive therapy for SARS-CoV-2 pneumonia

**DOI:** 10.1007/s15010-020-01522-4

**Published:** 2020-09-10

**Authors:** Valentina Marchese, Verena Crosato, Maurizio Gulletta, Filippo Castelnuovo, Graziella Cristini, Alberto Matteelli, Francesco Castelli

**Affiliations:** 1 Specialist Consultation Service ASST Spedali Civili; University Division of Infectious and Tropical Diseases, University of Brescia, ASST Spedali Civili Hospital, Brescia, Italy; 2 University Department of Infectious and Tropical Diseases, University of Brescia, ASST Spedali Civili Hospital, Brescia, Italy; 3 University Division of Infectious and Tropical Diseases, ASST Spedali Civili Hospital, Brescia, Italy; 4 University Division of Infectious and Tropical Diseases, University of Brescia, ASST Spedali Civili, Brescia, Italy; 5 University Division of Infectious and Tropical Diseases, University of Brescia, ASST Spedali Civili Hospital, Brescia, Italy

**Keywords:** *Strongyloides stercoralis*, Tocilizumab, SARS-CoV-2, Immunosuppression

## Abstract

**Background:**

SARS-CoV-2 pandemic has posed formidable public health and clinical challenges. The use of immunosuppressive agents, such as high dose corticosteroids and cytokine inhibitors (e.g., Tocilizumab) has been suggested to contrast the hyperinflammatory process involved in the pathogenesis of the severe disease, with conflicting evidence. Among the drawbacks of immunosuppressive therapy, the risk of reactivation of latent infections, including parasitic infestations, is to be considered.

**Case presentation:**

We report a case of a 59-year-old Italian patient treated with high dose intravenous dexamethasone and two intravenous doses of Tocilizumab for interstitial bilateral pneumonia associated with SARS-CoV-2 infection who developed itching, abdominal pain, and an increased eosinophil count. Stool examination confirmed the presence of *S. stercoralis* larvae. The patient was treated with a 4-day course of Ivermectin with full recovery.

**Discussion:**

We report the first case of *S. stercoralis* infection following an 11-day treatment with high-dose steroids and Tocilizumab for severe COVID-19. Clinicians should be aware of the risk of strongyloidiasis as a complication of the treatment for severe COVID-19.

## Background

The recent emergence of SARS-CoV-2 and its rapid spread throughout all continents has become a global concern [1]. Many studies have recently been conducted to identify the molecular pathway leading to alveolar damage in moderate and severe Coronavirus disease 2019 (COVID-19), and have shown the pivotal role of the hyperinflammatory response of the patients’ immune system to the virus in determining alveolar destruction [2]. This is the rationale for the use of corticosteroids, to counteract respiratory failure in severely ill patients, along with oxygen supply [3]. Clinical observational studies have described an improvement in clinical symptoms and oxygenation after steroid administration in patients with severe COVID-19 [4–7]. Moreover, one randomized clinical trial has been recently published to support a survival benefit (the RECOVERY trial) [8]. This controlled, open-label randomized trial provides evidence that treatment with dexamethasone at a dose of 6 mg once daily for up to 10 days reduces 28-day mortality in hospitalized patients with COVID-19 who are receiving oxygen supply, but not among those receiving no respiratory support. On the other hand, IL-6 has been implicated in the pathogenesis of the disease, causing what is known as “cytokine release syndrome” (CRS), contributing to the development of ARDS. Tocilizumab, an IL-6 inhibitor that has been approved for the treatment of rheumatoid arthritis, has been suggested to reduce ARDS-related complications in patients affected by COVID-19 [9, 10].

Previous experiences with tocilizumab suggest an increased risk of opportunistic and bacterial infection similar to anti-TNF-α agents. When using this drug, continuous clinical monitoring is recommended, as secondary infections might arise [11].

Strongyloidiasis is a parasitic disease widely distributed in tropical and subtropical regions [12]. It is mainly caused by *Strongyloides stercoralis* (seldom by other *Strongyloides* species), a soil-transmitted helminth that spread primarily through contaminated soil The presence of this helminth has been well documented in some temperate countries, especially in the past, like the Mediterranean basin [13].

Rhabditiform larvae are eliminated through the stool in soil, where they can develop either infective filariform larvae directly, or free-living adult male and female worms, which mate and develop eggs, rhabditiform larvae and eventually infective filariform larvae. Infective filariform (L3) larvae penetrate the human host skin and migrate to the small intestine, where they become adult female worms, which produce eggs via parthenogenesis and new rhabditiform larvae. These can either be eliminated through stool or can become infective filariform larvae, penetrating either the intestinal mucosa or the skin of the perianal area, resulting in autoinfection [14]. By this peculiar auto-infective cycle untreated cases can generate persistent, lifelong infections, and represent a risk factor for a potentially fatal hyperinfection syndrome or disseminated infection [15].

So far, only a few reports describe exacerbation of *S. stercoralis* infection in patients treated with either tocilizumab or anti TNF-α agents [16, 17].

## Case report

A 59-year-old woman born in Southern Italy was admitted to our ward in March 2020 after experiencing malaise, nausea, vomiting and fever lasting about a week. Chest x-ray showed bilateral basal interstitial pneumonia and SARS-CoV-2 RT-PCR in a oropharyngeal/nasal swab resulted positive. Since arterial pO2 was 57 mmHg, she was started on high-flow supplemental oxygen support. The patient reported chronic treatment with low dose prednisone for adult Still’s disease since 2010 and atenolol for hypertension.

Treatment with hydroxychloroquine, lopinavir/ritonavir, and dexamethasone was started together with enoxaparin prophylaxis. On the 5th day of hospitalization due to severe hypoxia and worsening of respiratory performance, she underwent non-invasive mechanical ventilation with continuous positive airway pressure (CPAP), which was continued for a total of 11 days. On day 7th she was treated with two doses of tocilizumab 8 mg/kg 12 h apart. Dexamethasone treatment was given at the dose of 20 mg/day for 5 days, followed by 10 mg/day for other 6 days. During the hospitalization, she presented an episode of atrial fibrillation, which was successfully reverted by amiodarone, and hyperglycemia, for which she started insulin-based treatment, later switched to oral hypoglycemic agents. Overall her clinical condition gradually improved, and she completed oxygen weaning on day 27th of hospitalization.

On day 25th her eosinophil absolute count (EAC) increased up to 5540 cell/µL and the patient reported abdominal pain and itching. Stool examination revealed the presence of rhabditiform larvae of *S. stercoralis*, while IFAT serology tested positive at a titre of 1:640. A 4-day oral treatment with ivermectin (200 mcg/kg) was administered, with a rapid decrease of eosinophil cell count and symptom improvement. She was discharged and a follow-up visit 1 month later was scheduled to check EAC, serology for *S. stercoralis* and stool examination.

The patient did not develop fever or worsening clinical condition concomitant to EAC rising. She denied travelling to tropical or subtropical areas and revealed recent moving to Lombardia region from Calabria region (Southern Italy). She reported repeated episodes of diffuse itching in the last 10 years, treated with topical steroids with partial improvement.

## Discussion

To date, no case of strongyloidiasis related to severe COVID-19 treatment has been reported. Nevertheless, based on the experiences from the use of steroids and tocilizumab in other diseases, it is conceivable that exacerbation of *S. stercoralis* infestation may occur [18]. Efficacy of immunosuppressive treatments for severe COVID-19 is still debated. In particular for tocilizumab, a recent meta-analysis did not show any additional benefit for patients with severe COVID-19 [19]. The authors concluded that further recommendations on tocilizumab should wait results from on-going clinical trials, due to the low quality of evidence of the available studies. Nevertheless, despite the promising preliminary data of RECOVERY trial on steroid administration [8], some concerns have been raised about the applicability of these results in different settings, such as in low-income, African countries [20]. Authors highlighted the risk of harms rather than benefits from steroid administration as a consequence of the different epidemiology of other infectious diseases, like tuberculosis or strongyloidiasis (which may be reactivated or worsened).

Strongyloidiasis is mainly an asymptomatic or mildly symptomatic disease [21], often only accompanied by a moderate increased EAC. In a recent study conducted in Northern Italy the prevalence of infection was 8% in Italian patients with EAC ≥ 500 cells/µL, especially in those born before 1947 and originating from rural areas surrounding the Po river, regardless of symptoms. The prevalence of strongyloidiasis was even higher (17%) in immigrants originating from endemic areas with eosinophilia [13]. Our patient had only recently moved to Lombardy: her 10-year history of itching suggests that she might have acquired the infection in Southern Italy.

In case of immunosuppression, strongyloidiasis can determine an hyperinfection syndrome or disseminated infection, with fatality rates up to 70–100% [15]. In *Strongyloides* hyperinfection syndrome an acceleration in the parasite life cycle leads to excessive reproduction rates within the traditional reproductive sites of the worm (skin, guts and lungs). The number of larvae increases in stools and/or sputum along with clinical manifestations to the respiratory, gastrointestinal system and peritoneum. Disseminated strongyloidiasis is a severe infection which results from massive dissemination to body districts the parasite does not normally reach and colonise, such as the liver, heart, brain and the urinary tract [22].

Hyperinfection or disseminated strongyloidiasis are rarely reported in patients treated with tocilizumab. To date, only one case report described the onset of haemorragic alveolitis following combined steroid and tocilizumab treatment [17], while sporadic cases following anti TNF-α or high dose corticosteroid treatments have been reported [16, 23].

Given these high fatality rates, a screening process should be performed when using such therapies in patients with risk of exposure to *Strongyloides*, and, if diagnostic test is not available, pre-emptive treatment with ivermectin should be considered [24]. The use of Tocilizumab for COVID-19 is limited to a short-term treatment course, which includes a few doses (generally 2 or 3). If compared to the long treatment course which is licensed for rheumatological diseases, the risk for opportunistic disease reactivation should be limited, though no specific studies have yet been conducted.

Our patient did not present increased EAC on admission, which suggests that the worsening of patient’s strongyloidiasis was associated with the use of tocilizumab and high-dose corticosteroids. Our patient did not develop an hyperinfection syndrome or a disseminated infection, possibly due to the rapid detection of the infection and its prompt treatment. It is also possible that the leukocyte formula alterations in the course of COVID-19 could have masked a pre-existing mild hypereosinophilia, as a consequence of the hyperinflammatory response to SARS-CoV-2 infection.

A dedicated strategy based on epidemiological risk stratification has been recently proposed to prevent *Strongyloides* hyperinfection/disseminated infection for COVID-19 patients undergoing steroids [25]. In inpatient clinical settings, presumptive ivermectin treatment is proposed for at-risk patients who initiate or are candidates for steroids, as well as in case of invasive gram-negative rod infection while waiting for diagnostic tests. Even in outpatient setting, in presence of risk factor for strongyloidiasis, a presumptive treatment (usually one dose) should be considered, if it is not contraindicated and serology is not available.

In confirmed uncomplicated infection the efficacy of a single-dose treatment has been well established [26], longer treatment being suggested in hyperinfection/dissemination [27]. In our case the underlying immunosuppressive treatment prompted us to adopt a treatment longer than that proposed for uncomplicated infection.

In conclusion, we report the first case of strongyloidiasis following high-dose steroid and tocilizumab treatment for severe COVID-19. Risk assessment for strongyloidiasis should be performed for people who live or have visited areas, where the organism is endemic. Similarly, we suggest considering this helminth in case of unexplained appearance of hypereosinophilia. When a prompt diagnosis is not feasible, due to the urgency of treatment and the risk of fatal outcome either for COVID-19 or *Strongyloides* hyperinfection/dissemination syndrome, empirical pre-emptive single-dose ivermectin therapy must be considered.
